# hBD-2 is downregulated in oral carcinoma cells by DNA hypermethylation, and increased expression of hBD-2 by DNA demethylation and gene transfection inhibits cell proliferation and invasion

**DOI:** 10.3892/or.2014.3260

**Published:** 2014-06-12

**Authors:** YOSHITAKA KAMINO, YOSHIHITO KURASHIGE, OSAMU UEHARA, JUN SATO, MICHIKO NISHIMURA, KOKI YOSHIDA, TOSHIYA ARAKAWA, HIROKI NAGAYASU, MASATO SAITOH, YOSHIHIRO ABIKO

**Affiliations:** 1 Division of Oral Medicine and Pathology, Department of Human Biology and Pathophysiology, School of Dentistry, Health Sciences University of Hokkaido, Hokkaido 061-0293, Japan; 2 Sapporo Oral and Maxillofacial Surgery Clinic, Sapporo 004-0051, Japan; 3 Division of Oral and Maxillofacial Surgery, Department of Human Biology and Pathophysiology, School of Dentistry, Health Sciences University of Hokkaido, Hokkaido 061-0293, Japan; 4 Division of Pediatric Dentistry, Department of Oral Growth and Development, School of Dentistry, Health Sciences University of Hokkaido, Hokkaido 061-0293, Japan; 5 Division of Disease Control and Molecular Epidemiology, Department of Oral Growth and Development, School of Dentistry, Health Sciences University of Hokkaido, Hokkaido 061-0293, Japan; 6 Division of Biochemistry, Department of Oral Biology, School of Dentistry, Health Sciences University of Hokkaido, Hokkaido 061-0293, Japan

**Keywords:** β-defensin, antimicrobial peptide, epigenetics, hypermethylation, gene transfection, oral carcinoma

## Abstract

Human β-defensin-2 (hBD-2) is a type of epithelial antimicrobial peptide. The expression level of hBD-2 mRNA is lower in oral carcinoma cells (OCCs) than in healthy oral epithelium. Yet, it is still unknown how hBD-2 expression is downregulated in OCCs. The present study investigated DNA hypermethylation of hBD-2 in OCCs and the effect of the demethylation and increased expression of hBD-2 on cell proliferation and invasion. Six different types of oral carcinoma cell lines (OSC-19, BSC-OF, SAS, HSC-2, HSC-4 and HSY) and normal oral keratinocytes (NOKs) were used. The expression levels of hBD-2 in all OCCs were significantly lower than that in the NOKs. Treatment with DNA methyltransferase inhibitor, 5-aza-dC, at the concentration of 50 μM significantly induced upregulation of expression of hBD-2 in the OCCs. Using methylation-specific PCR, DNA hypermethylation was observed in all OCCs. These results suggest that DNA hypermethylation is, at least in part, involved in the decreased expression of hBD-2 in OCCs. We examined the effect of 5-aza-dC on the cell proliferation and invasive ability of OCCs. The cell invasion assays showed that the number of OCCs treated with 5-aza-dC on the filters was significantly lower than that of the controls. We examined whether increased expression of hBD-2 generated by gene transfection inhibited the proliferation and invasion of SAS cells. The number of SAS cells exhibiting increased expression of hBD-2 on the filters in the invasion assay were significantly lower on day 7 when compared with the control. hBD-2 may function as a tumor suppressor. Increased expression of hBD-2 induced by demethylation or increased expression generated by gene transfection may be useful therapeutic methods for oral carcinoma.

## Introduction

The human defensins are small cationic antimicrobial peptides that are classified into two classes, α-and β-defensins. The α-defensins are expressed in neutrophils and in Paneth cells in the intestinal crypt. Human β-defensins (hBDs) are mainly produced by the epithelial cells of numerous organs including the skin, lung, kidney, pancreas, uterus, eye and nasal and oral mucosa. hBD-1, -2 and -3 among the many types of hBDs have been well researched. hBD-1 is constitutively expressed by various tissues and may be modulated by inflammation. hBD-2 and hBD-3 are expressed by cells upon stimulation with proinflammatory cytokines such as interleukin (IL)-1β, tumor necrosis factor (TNF)-α and interferon (IFN)-γ and by microorganisms (1,2). Evidence has recently suggested the involvement of hBDs in carcinogenesis as well as their antimicrobial activity (3,4). The expression levels of hBD-1 and hBD-2 mRNA were found to be lower in oral carcinoma than in healthy oral epithelium (5–7). Overexpression of β-defensin-1 in renal carcinoma cells induced apoptotic cell death, implying that hBD-1 may function as a tumor-suppressor gene (8). Decreased expression of hBD-1 in OCCs may be due to their single nucleotide polymorphisms (SNPs) (7), and may play a role in the development of oral carcinoma (6). It is, however, still unclear how hBD-2 expression is downregulated in oral carcinoma. Gene polymorophisms and mutations, loss of heterozygosity, and epigenetic modifications are involved in the aberrant transcriptional levels in malignant tumors (9,10). Neither directly connected SNPs, common gene mutations nor epigenetic modifications of hBD-2 have been shown to date. DNA methylation and histone modifications are two major mechanisms of epigenetic modifications in human cells (11). DNA methylation is characterized by the addition of a methyl group to cytosines within CpG regions. DNA hypermethylation leading to a decrease in gene transcriptional levels frequently occurs in tumor-suppressor genes in malignant tumors. The decreased expression of tumor-suppressor genes by DNA hypermethylation promotes malignant transformation (9–11).

Therefore, we hypothesized that DNA hypermethylation is involved in the decreased expression of hBD-2 in OCCs, and hBD-2 may play a role in the development of OCCs. The present study investigated the DNA hypermethylation of hBD-2 in OCCs and the effect of demethylation and increased expression of hBD-2 on cell proliferation and invasion.

## Materials and methods

### Cell cultures

Normal human oral keratinocytes (NOKs) were isolated from healthy gingival tissue overlying the impacted third molar of an adult human. Briefly, explants of the healthy gingival tissue obtained from the third molar surgical extraction were cultured in Dulbecco’s modified Eagle’s medium (DMEM; Sigma-Aldrich, St. Louis, MO, USA) containing 10% fetal bovine serum (FBS; Gibco, Invitrogen Corp., Carlsbad, CA, USA) and antibiotics (100 U/ml penicillin; 200 μg/ml streptomycin; 5 μg/ml amphotericin B; Sigma-Aldrich) and 30 mg/ml Fungizone (Bristol-Myers Squibb, Tokyo, Japan) at 37°C in a humidified atmosphere of 95% air and 5% CO_2_. Outgrowth developed after 2 or 3 weeks of incubation. The two cell types were separated into epithelial populations that were more or less resistant to detachment with 10% dispase (Godo Shusei Co., Ltd., Tokyo, Japan). The cells that were less resistant were detached, removed from the rest of the cell population and discarded. The attached cells were cultured. The separation procedure was repeated two or three times so as to remove any fibroblasts. NOKs were grown in Keratinocyte Basal Medium (KBM; Lonza Walkersville, Inc., Walkersville, MD, USA) supplemented with 7.5 mg/ml bovine pituitary extract (Lonza), 0.1 μg/ml hEGF (Lonza), 5 mg/ml insulin (Lonza), 0.5 ml aqueous solution of gentamicin sulfate (Lonza) and amphotericin-B.

Five different types of oral squamous cell carcinoma cell lines (OSC-19, BSC-OF, SAS, HSC-2 and HSC-4) and a human salivary adenocarcinoma cell line (HSY) were incubated with DMEM supplemented with 10% FBS, 100 U/ml penicillin and 200 μg/ml streptomycin.

To examine the role of hBD-2 methylation in OCCs, the cells were treated with 50 μM of the DNA demethylating agent, 5-aza-2′-deoxycytidine (5-aza-dC; Sigma-Aldrich) or the same volume of DMSO as control for 24 and 48 h.

### Quantitative real-time RT-PCR

The expression levels of hBD-2 mRNA in the cultured cells were analyzed by quantitative real-time RT-PCR. Total RNA was extracted from cells by the acid guanidine thiocyanate/phenol-chloroform method using TRIzol (Invitrogen). The RNA was reverse transcribed (SuperScript Reverse Transcriptase; Invitrogen), according to the manufacturer’s instructions using oligo(dT)_12–18_ primers (Invitrogen). Specific primer and probe sets for hBD-2 and GAPDH were purchased from Applied Biosystems (Foster City, CA, USA) (hBD-2; Hs00823638_m1, GAPDH; Mm99999915_g1). The reaction mixture was prepared using TaqMan Universal PCR Master Mix (Applied Biosystems) with primer, probe sets and RT products. Real-time PCR was performed using a GeneAmp 5700 Sequence Detection System instrument and software (Applied Biosystems). The expression level of hBD2 mRNA was standardized against GAPDH mRNA. The relative expression of hBD-2 mRNA was calculated using the 2^−ΔΔCt^ method as described by Saitoh *et al* (12). Data are expressed as the ratio of hBD-2 mRNA to GAPDH mRNA.

### Methylation-specific polymerase chain reaction method

In order to analyze CpG island hypermethylation of hBD-2, the methylation profiles were assessed using a methylation-specific PCR (MSP) method, similar to that reported by Herman *et al* (13). Briefly, DNA was extracted from the cultured cells with DNeasy Blood & Tissue kit (Qiagen, Stanford, CA, USA) according to the manufacturer’s protocol. The DNA was purified using a phenol/ethanol method. MSP distinguishes unmethylated from methylated alleles in a given gene based on sequence changes produced after bisulfite treatment of DNA, which converts unmethylated (but not methylated) cytosines to uracil, and subsequent PCR using primers designed for either methylated or unmethylated DNA. The primer sequences are listed in Table I. PCR was performed using an amplification kit (AmpliTag Gold; Applied Biosystems) and a thermal cycler (Takara PCR Thermal Cycler MP; Takara, Osaka, Japan). Each PCR product was loaded directly onto non-denaturing 2% agar gels. As a positive control for methylation, CpGenome™ Universal Methylation DNA (Millipore, Billerica, MA, USA) was used.

### Increased expression of hBD-2

hBD-2 containing the entire coding region was amplified by PCR using NOK cDNA as a template. The primer sets used were 5′-CGCGGATCCATGA GGGTCTTGTAT-3′ (forward) and 5′-CCGCTCGAGTCAT GGCTTTTTGCA-3′ (reverse) for cDNA amplification. The amplified products were inserted into *Bam*H1 and *Xho*I cloning sites of the pcDNA 6/His C (Invitrogen). The inserted hBD-2 sequence was confirmed with a standard DNA sequencing method. SAS cells were transfected with the hBD-2-inserted plasmid using the lipofection method (Effecten™; Qiagen, Hilden, Germany). Transfected clones were selected in 5 μg/ml Blasticidin S (Invitrogen). As a control, the pcDNA 6/His C mock vector was transfected into the same cell lines.

### Cell proliferation assay

Control and hBD-2-transfected SAS cells were seeded at a concentration of 2×10^5^ cells/ml on 96-well plates in DMEM containing 10% FBS and were cultured for 24 h. After 24 and 48 h of incubation of the cells, XTT assays were performed. XTT solution (1 mg/ml XTT in 80 ml; Sigma) and 0.025 mM phenazine methosulphate were added to the cells in a 96-well microplate. After a 3-h incubation, the optical density (OD) at 490 nm was measured.

### Cell invasion assay

Cell migration was assessed in 6-well Transwell units with an 8-μm pore polycarbonate filter membrane coated with Matrigel™ (BD Biosciences, San Jose, CA, USA). Control and hBD-2-transfected SAS cells in serum-free DMEM at a density of 2×10^5^ cells/ml were loaded on the membrane in the upper chamber. The upper chambers were gently inserted into DMEM supplemented with 10% FBS in the lower chamber. After 7 days, non-migrating cells on the upper side of the membrane were removed by a sterile cotton swab, and the remaining invaded cells on the lower side of the membrane were fixed and stained with hematoxylin. The number of cells in three fields per well were counted under a microscope.

### Statistical analysis

Data from the real-time RT-PCR, cell proliferation assay and cell mobility assay were analyzed using the Student’s t-test (2-tailed). A P-value of <0.05 was considered to indicate a statistically significant result.

## Results

**Figure 1:**
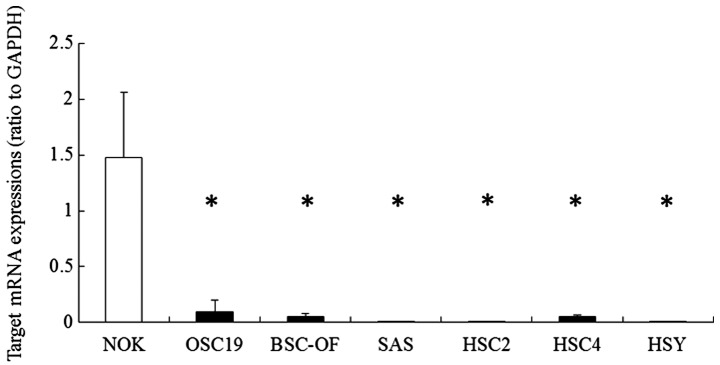
Expression levels of hBD-2 mRNA in NOKs and SCC cell lines. The expression levels of hBD-2 in OSC-19, BSC-OF, SAS, HSC-2 and HSC-4 and HSY cell lines were significantly lower than that in the NOKs (^*^P<0.05).

**Figure 2:**
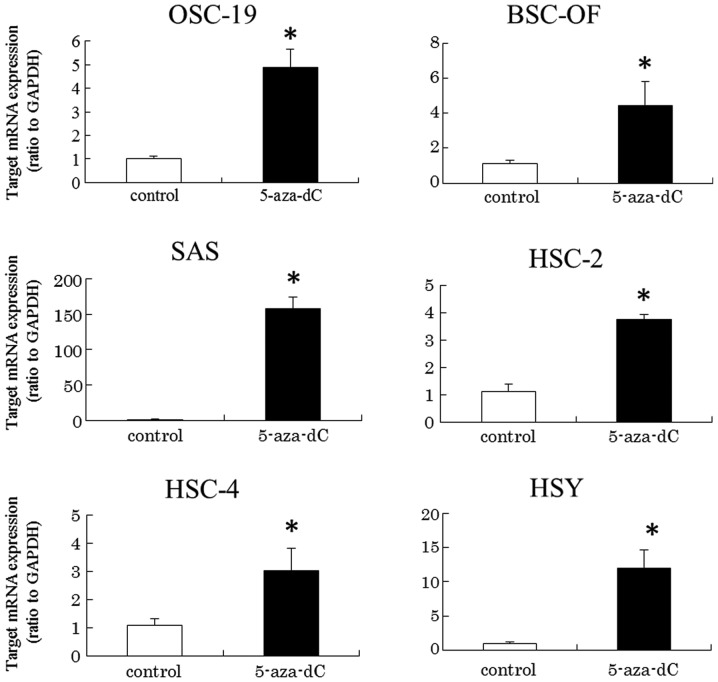
Expression levels of hBD-2 in SCC cell lines stimulated with 50 μM of 5-aza-dC. Expression levels of hBD-2 in the OSC-19, BSC-OF, SAS, HSC-2, HSC-4 and HSY cells treated with 50 μM of 5-aza-dC were significantly higher than the level in each control (^*^P<0.05).

**Figure 3:**
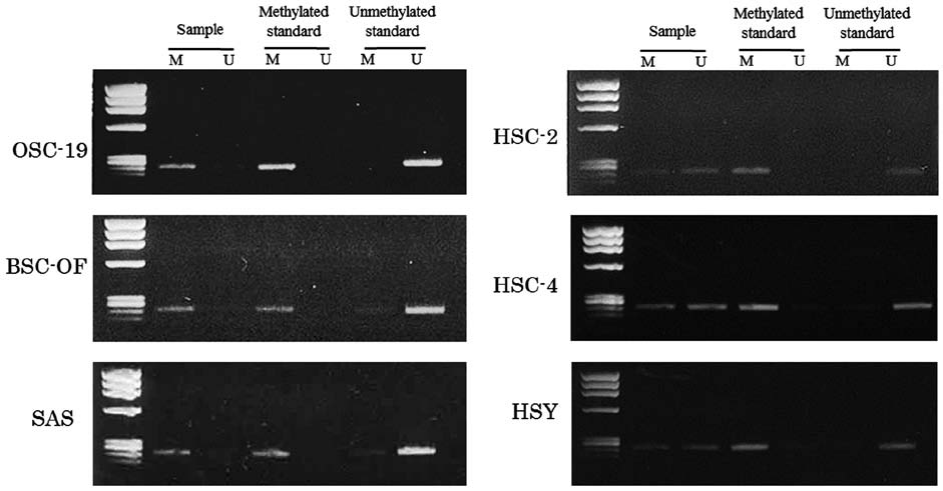
Results of the methylation-specific PCR in the oral SCC cell lines. OCCs were analyzed by MSP after bisulfite conversion of the DNA. We obtained a strong PCR product using the methylated primer in OSC-19, BSC-OF and SAS cells, and a weaker band with the methylated primer as shown in the left images. In contrast, the DNA was amplified to similar PCR products with both the methylated and unmethylated primers in HSC-2, HSC-4 and HSY cells, as shown in the right images. M, methylated; U, unmethylated.

First, we observed the expression of hBD-2 mRNA in the OCCs by quantitative RT-PCR. The expression levels in OCCs were compared with that in the NOKs. The expression levels of hBD-2 mRNA in the OCCs (OSC-19, BSC-OF, SAS, HSC-2, HSC-4 and HSY) were significantly lower than that in the NOKs (Fig. 1). In order to examine whether DNA hypermethylation is involved in the transcriptional levels, cells were treated with DNA methyltransferase inhibitor, 5-aza-dC, at the concentration of 50 μM. The inhibitor significantly induced upregulated expression of hBD-2 in the OCCs (Fig. 2). The results indicate that DNA hypermethylation may be involved in the downregulated expression of hBD-2 mRNA in the OCCs. The DNA hypermethylation in the OCCs was examined using an MSP method. The primers for the MSP method were designed in the promoter region lower than −1,500 kbp. The data for the MSPs are illustrated in Fig. 3. Bisulfite treatment converts unmethylated cytosines to uracil, which shows a visible PCR product when amplified by unmethylated primers (lane U). No conversion of the methylated cytosines was shown as a visible PCR product when amplified by methylated primers (lane M). The product visualized only in lane M, both in U and M, and only in U indicates complete, partial and no hypermethylation, respectively. Complete hypermethylation was observed in OSC-19, BSC-OF and SAS cells, and partial hypermethylation was observed in HSC-2, HSC-4 and HSY cells (Fig. 3). These results confirmed that DNA hypermethylation was, at least in part, involved in the decreased expression of hBD-2 in the OCCs.

**Figure 4:**
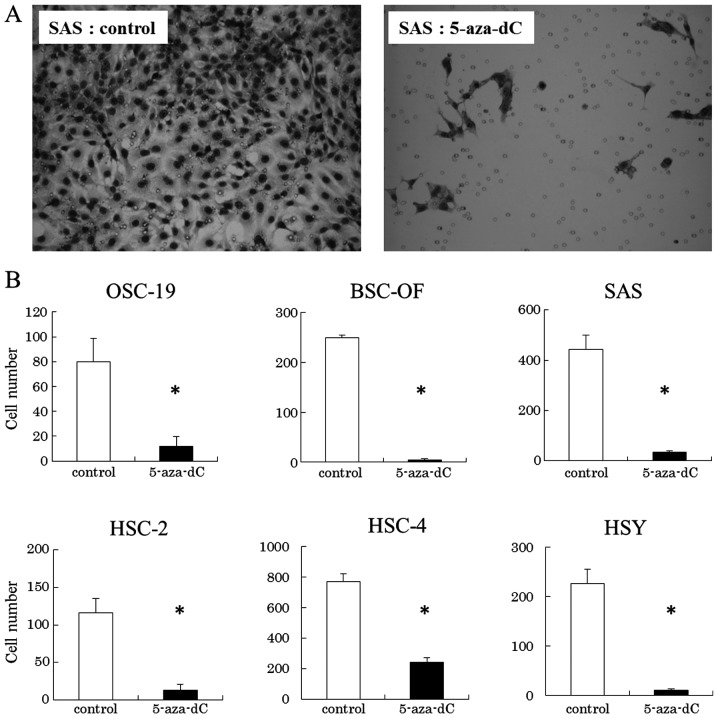
Cell migration assay. The number of cells treated with 5-aza-dC that migrated to the filters in the migration assay were significantly lower than the control (^*^P<0.05).

**Figure 5:**
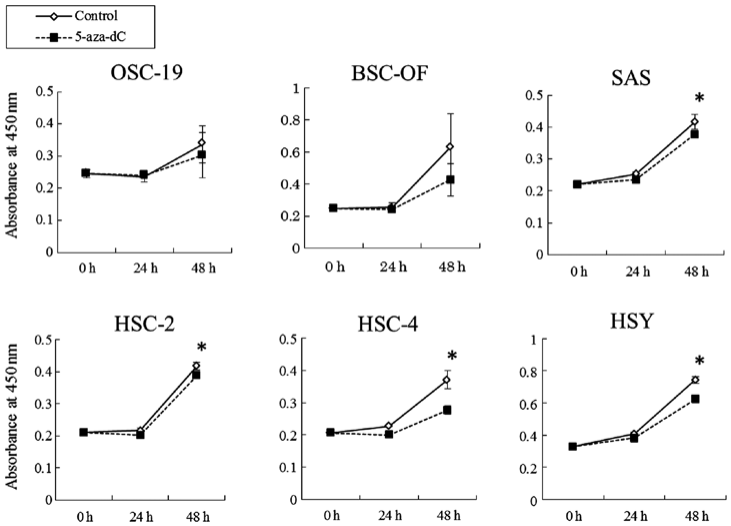
Effect of 50 μM of 5-aza-dC on the proliferation of oral SCC cell lines (XTT assay). The cell growth rates of the SAS, HSC-2, HSC-4 and HSY cells treated with 5-aza-dC were significantly lower than that of the control cells (^*^P<0.05).

**Figure 6:**
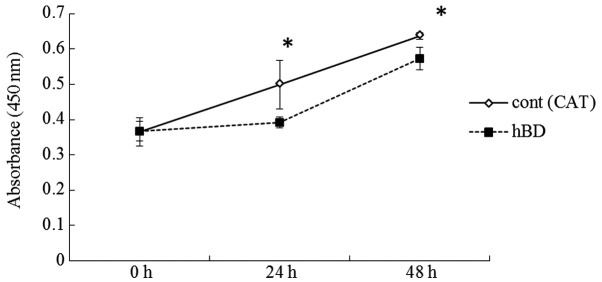
Cell growth assay (XTT assay). The cell growth rate of hBD-2-transfected SAS cells was significantly lower than that of the control cells. (^*^P<0.05).

**Figure 7:**
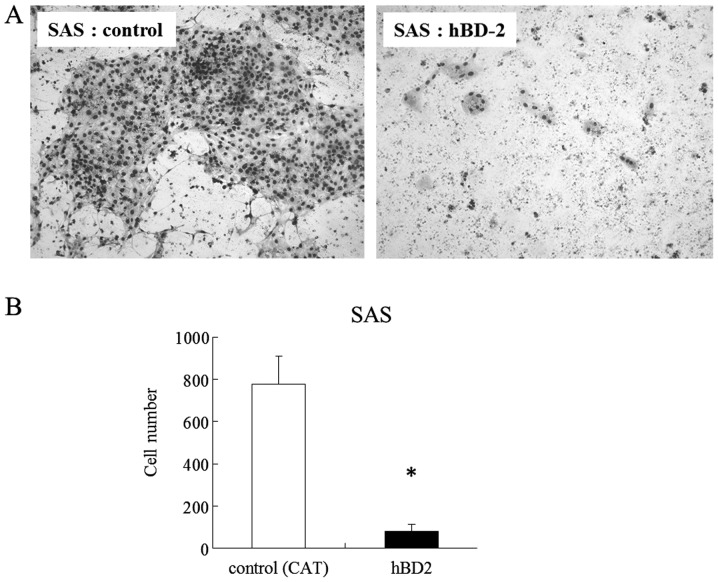
Cell migration assay. The number of migrating cells exhibiting increased expression of hBD-2 on the filters in the migration assay was significantly lower than that of the control (^*^P<0.05).

In order to ascertain whether the decreased expression of hBD-2 is involved in cell proliferation and invasion implying malignant potential, we examined whether 5-aza-dC that induced upregulated expression of hBD-2 in cells could inhibit the cell proliferation and invasion of OCCs. The growth rates of SAS, HSC-2, HSC-4 and HSY cells treated with 5-aza-dC at the concentration of 50 μM were significantly lower than that of the controls. No significant differences were observed between the OSC-19 or BSC-OF cells treated with 5-aza-dC and the controls (Fig. 4). The cell invasion assays showed that the number of OCCs treated with 5-aza-dC on the filters were significantly less than that of the controls (Fig. 5). We examined whether increased expression of hBD-2 generated by gene transfection inhibited the cell proliferation and invasion. Since expression level of hBD-2 in SAS cells was lowest among the OCCs, increased expression of hBD2 in SAS was generated by the hBD-2 gene. The number of cells exhibiting increase expression of hBD-2 were significantly lower at 24 and 48 h than the control (Fig. 6). The number of cells exhibiting increased expression of hBD-2 that migrated to the filters in the invasion assay were significantly lower on day 7 than the control (Fig. 7).

## Discussion

The present study first demonstrated that DNA hypermethylation is involved in decreased expression of hBD-2 in OCCs, and that increased expression of hBD-2 inhibited OCC proliferation and invasion. Thus, hBD-2 is likely to be a putative tumor-suppressor gene.

The expression levels of hBD-2 mRNA were found to be lower in OCCs than that in healthy oral epithelium (5,7,14). The present study showed that the expression level of hBD-2 in 6 types of OCCs was lower than that in NOKs which supported the previous data. The aberrant expression of certain types of genes are frequently observed in cancers. The aberrant expression of tumor-suppressor genes such as p14, p15, p16 and p53 is often involved in carcinogenesis (9,15). Several point mutations frequently occur in p53. The half-life of mutant p53 is longer than that of the wild-type, resulting in the accumulation of mutant p53. The accumulation leads to overexpression of p53 (16). The p14, p15 and p16 genes are often downregulated induced by DNA hypermethylation in malignant cells. DNA hypermethylation in p14, p15 and p16 was frequently found in OCCs (9,10). Therefore, we examined whether the downregulation of the expression of hBD-2 involves DNA hypermethylation. Treatment with 5-aza-dC, a DNA methyltransferase inhibitor, significantly induced the upregulation of the expression of hBD-2 in the OCCs. The MSP method confirmed the DNA hypermethylation of hBD2 in the OCCs. These results suggest that DNA hypermethylation is involved in the decreased expression of hBD-2 in OCCs.

As mentioned above, tumor-suppressor genes such as p14, p15 and p16 are often downregulated in OCCs, induced by DNA hypermethylation. The downregulation is possibly linked to OCC development (9,10). hBD-2 may function as a tumor-suppressor since downregulated expression of hBD-2 is caused by DNA hypermethylation in OCC. In order to clarify this speculation, we examined whether increased expression of hBD-2 in the OCCs affects their cell proliferation and invasion capacities. Treatment with 5-aza-dC inhibited the cell proliferation and invasion of OCCs as well as induced upregulated expression of hBD-2. DNA methyltransferase inhibitors including azacytidene, decitabine and zabularine have been widely applied for experimental cancer therapy (17). Our data confirmed that 5-aza-dC, a type of azacytidene, may be a therapeutic agent for OC. It was unclear whether the inhibition of cell proliferation and invasion were due to the effect of 5-aza-dC or the increased expression of hBD-2. We observed the effect of increased expression of hBD-2 on OCC proliferation and invasion. Since the expression level of hBD-2 in SAS cells was lowest among the OCCs, we used SAS cells transfected by the hBD-2 gene to increase the expression of hBD-2. Increased expression of hBD-2 in the SAS cells inhibited their proliferation and invasion. The inhibitory effect of 5-aza-dC on OCC proliferation and invasion may be partially via increased expression of hBD-2. The effects of the hBD-2 peptide on carcinoma cell lines have been previously reported (18–20). hBD-2 was found to have little effect on the proliferation of carcinoma cells (18). The hBD-2 peptide was reported to promote and inhibit the proliferation of carcinoma cells at low and high concentrations, respectively (19). hBD-2 promoted the proliferation of carcinoma cells, implying its function as a proto-oncogene (20). The effects of hBD-2 on carcinoma cells may be dependent on the concentration of the hBD-2 peptide and the type of cells. Unlike these previous reports that showed an effect of the exogenous hBD-2 peptide on the cells, we observed the effect of endogenous hBD-2 expression on carcinoma cells. The increased endogenous expression of hBD-2 clearly inhibited their own cell proliferation and invasion. The mechanism of the inhibitory effect of endogenous hBD-2 on carcinoma cells is unknown. The inhibitory effect of exogenous hBD-2 peptide on cell proliferation might be via G1/S arrest and pRB gene expression (19). The increased expression of hBD-1 in renal carcinoma cells generated by hBD-1 gene transfection inhibited proliferation of their own cells and resulted in caspase-3-mediated apoptosis (8). The overexpression of hBD-1 in a keratinocyte cell line, HaCaT, induced by gene transfection promoted differentiation of the cells. On the other hand, the overexpression of hBD-2 in the HaCaT cells did not affect their differentiation (21). Although no increased expression of hBD-2 was observed in the HaCaT cells under the conditions required for differentiation (22), the expression of hBD-2 was upregulated in other types of keratinocytes by stimulation with keratinocyte differentiation (23,24). An inhibitory effect of increased expression of hBD-2 on the proliferation and invasion of OCCs was noted which may be due to the response to stimulation of OCC differentiation. Further investigations are needed to clarify the inhibitory mechanism of cell proliferation and invasion by endogenous hBD-2 expression. The inhibitory effect caused by increased endogenous expression of hBD-1 on the carcinoma cells suggests that hBD-1 is a potential tumor-suppressor gene. Further investigations will be needed to verify this speculation.

In conclusion, we found involvement of DNA hypermethylation in the decreased expression of hBD-2 in oral carcinoma cell lines. Increased expression of hBD-2 in the cells inhibited their proliferation and invasion. Therefore, hBD-2 functions as a tumor suppressor. Increased expression of hBD-2 induced by demethylation or by gene transfection may be a useful therapeutic method for oral carcinoma.

## Figures and Tables

**Table I:** hBD2 primer design for MSP.

Primers 241 bp	Primer sequence (5′-3′)
Methylation primers (M)	F: TTGGTTTGTTAGGAATTAGGGTTT
	R: CCATCCC**G**AACACTCAAAA
Unmethylation primers (U)	F: TTGGTTTGTTAGGAATTAGGGTTT
	R: CCATCCC**A**AACACTCAAAAA

MSP, methylation-specific polymerase chain reaction. Bold letters indicate a different sequence in the primers between M and U.
